# Investigation of the Effect of Folic Acid Based Iron Oxide Nanoparticles on Human Leukemic CCRF-CEM Cell Line 

**Published:** 2013-04-22

**Authors:** M Mehrabi, A Javid, A Hashemi, S Rezaei-Zarchi

**Affiliations:** 1 Genetics Group, Jehad Daneshgahi University, Yazd, Iran.; 2 Department of Biochemistry, Institute of Biochemistry and Biophysics, University of Tehran, Tehran, Iran.; 3 Department of Pediatrics, Hematology, Oncology and Genetics Research Center, Shahid Sadoughi University of Medical Sciences and Health Services,Yazd, Iran.; 4 Department of Biology, Payame Noor University, Yazd, Iran.

**Keywords:** Folate, Blood cancer, Nanoparticles

## Abstract

**Background:**

Nanoparticulate drug delivery systems have attracted significant attention in the field of cancer nanotechnology. This study determines the effect of folate-based Fe_2_O_3 _nanoparticles. This study aimed to decorate nanoparticles with folate (FA), a molecular ligand for ‘active’ targeting of cancerous cells and the application of modified-nanoparticles in cancer treatment.

**Materials and Methods:**

The nanoparticles were prepared by a solvent evaporation and emulsification cross-linking method and anticancer activity of agent was evaluated on CCRF CEM cells, derived from human blood cancer samples.

**Results:**

The physicochemical properties of the nanoparticles were characterized by various techniques, and uniform nanoparticles with an average particle size of 110±15 nm were obtained. Cytotoxicity tests showed that the SPIO-FA had higher cell toxicity, and confocal microscopy analysis confirmed excellent cellular uptake efficiency.

**Conclusion:**

These results indicate that FA based SPIO-NPs have potential uses as anticancer drug carriers and also have an enhanced anticancer effect.

## Introduction

The enzyme glucose-6-phosphate dehydrogenase even with the biological and pharmaceutical advancement that the scientific community has achieved, cancer is still, without doubt, one of the biggest killers worldwide. The current focus in the development of cancer therapies is on targeted drug delivery to provide therapeutic concentrations of anticancer agents at the site of action and spares the normal tissues. Numerous investigations have shown that both tissue and cell distribution profiles of anticancer drugs can be controlled by their entrapment in submicronic colloidal systems (nanoparticles). The rationale behind this approach is to increase antitumor efficacy, while reducing systemic side-effects. Nanoparticulate drug delivery systems have attracted significant attention in the field of cancer nanotechnology. Over the past decade, various self-assembled nanoparticulate carriers such as liposomes, polymeric micelles, and nanoparticles have been widely explored to selectively deliver anti-cancer agents to tumor tissues for effective cancer therapy (3). Swellable hydrophilic polymer nanoparticles, have recently gained much interests as promising nanoparticulate carriers due to their nanoscale size (50–200 nm) and high stability favorable for intravenous and intracellular drug delivery (4). They also provide an aqueous interior space for incorporation of various bioactive macromolecules such as proteins. Folic acid (FA) has recently emerged as a prominent targeting moiety, capable of interacting with cells expressing the folate receptor (FR) (Low and Kularatne, 2009). FR consists of a high affinity folate binding protein (FBP) attached to the membrane through a glycosylphosphatidyl-inositol anchor. It is over-expressed in blood carcinomas and other human tumors and has little expression in normal tissues. This provides tumor cells with increased amounts of the FA, which is essential for DNA synthesis and seems to aid in aggressive tumor growth. In patients with blood cancer, the over expression of FR isoform *a* correlates with a higher histological grade and more advanced stage of the disease. The differential expression of FR in blood and other cancers makes it an attractive marker and target molecule for diagnosis and therapy of the disease (Low et al., 2008). Several folate-conjugated drugs have reached clinical evaluation stage. The site-specific delivery of drugs to the tumors using FR can be enhanced using high capacity carriers that can simultaneously incorporate multiple drug molecules into one particle and target them to the disease sites (Xia and Low, 2010). Here, we demonstrate the successful synthesis of FA decorated magnetite nanoparicles. The anticancer effect of FA-MNPs was evaluated against the human blood cancer CCRF CEM cells.

## Materials and Methods

**Chemicals**

Cell culture reagents were from Life Technologies, Inc. (Grand Island, NY). Doxorubicin and folic acid (FA) were purchased from Sigma-Aldrich Chemical Co. (St. Louis. Missouri, USA). RPMI-1640 medium and all the additives were purchased from GIBCO Co. (Grand Island, NY, USA). The blood cancer cell lines, CP70 and C30, were purchased from Pasteur Institute, Tehran, Iran. All other chemicals were obtained through standard suppliers. 

**Synthesis of folate loaded magnetite NPs**

MNPs (Fe_3_O_4_) were prepared by co-precipitation technique, with some modifications in the previously reported method (Karen et al., 1997; Xu and Du, 2003). Firstly, 5.41 g of FeCl_3_·6H_2_O (99% purity) and 1.99 g FeCl_2_·4H_2_O (99% purity) were dissolved in 100 ml of distilled water in a three-necked flask. FA was activated with EDC in double-distilled water (pH 7.4) by stirring it for 5 minutes in the dark and then allowed to react with the solution of iron chlorides. Pressurized air was supplied to the above solution to oxidize Fe^2+^ to Fe^3+^ for the formation of magnetite (Fe_3_O_4_) (24-26). Change in the color of solution to dark brown to black, due to the precipitation of Fe_3_O_4_, indicated the formation of bare and FA-MNPs (Du, 2003; Qi et al., 2004). The TMAOH (tetramethyl ammonium hydroxide) was used as the surfactant, in preparing the bare MNPs to maintain the aqueous solution of bare NPs in the state of colloidal suspension. The supernatant was discarded and the resulting precipitate was collected with strong magnet and rinsed thrice with distilled water to remove excess NH_4_OH. FA-MNPs were purified using PD-10 desalting columns, thoroughly dialyzed against double-distilled water (MWCO 3.5 kDa) and lyophilized. 

**Characterization of synthesized NPs**

Size and surface morphology of the synthesized NPs was characterized with the help of Transmission Electron Microscope (TEM; H-7600, Hitachi High-Technologies Corporation, Tokyo, Japan). A dynamic light-scattering spectrometer (DLS-7000AL, Otsuka Electronics, Japan) was used to determine the average diameters of the bare and the coated NPs. The magnetization measurements were carried out at room temperature using a vibrating sample magnetometer (VSM, Oxford Instruments, UK), with the magnetic field rage of –1 to +1 Tesla (T). The presence of FA-coating onto the surface of MNPs was studied by wavelength-dependent data of transmittance, obtained for the powdered samples of bare and FA-MNPs, pressed into KBr pellets. The experiment was carried out using FTIR Spectrophotometer (Model 8300, Shimadzu Corporation, Tokyo, Japan) at 4000 to 400 cm^−1^. The crystallographic state of bare and HP-SPIO NPs was determined by XRD (JDX -8030). 

**Cell lines and culture conditions**

CCRF CEM cells, derived from human blood cancer samples, were cultured in RPMI-1640 medium containing 10% (v/v) heat-inactivated fetal calf serum (FCS), 100 U/mL penicillin, and 100 μg/mL streptomycin at 37 °C in a humidified 5% CO_2_ incubator.

**Cell proliferation assay**

An SPIO-FA solution was diluted with PBS solution to give a final concentration of heparan from 10-200 µM. Human blood cancer CCRF CEM cells were seeded in a 24-well plate at a density of 5×10^3^ cells/well and grown in RPMI-1640 medium supplemented with 10% (v/v) fetal calf serum for 24 h at 37 ^o^C. The cells were then incubated with the culture medium alone (control), with SPIO- FA for 3 days at 37 ^o^C. The number of viable cells was determined by the CCK-8 cell viability assay, which depends on the mitochondrial dehydrogenase activity inside the cells.

**Evaluation of cellular uptake and apoptosis-inducing effect of SPIO-FA complex**

Fluorescein-conjugated heparan was prepared by conjugating fluorescein-5-maleimide to thiolated heparan. In brief, 5 mg of thiolated heparan was reacted with 0.1 mg of fluorescein-5-maleimide in 0.1 M phosphate buffer (pH 7). The solution was dialyzed (Mw cutoff of 6 kDa) and then lyophilized. Using the fluorescent heparan, fluorescein-conjugated SPIO-FA complex was prepared by the methods described above. CCRF CEM cells were plated over a cover slide on a six-well plate at a density of 2×10^5^ cells/well and cultivated for 24 h at 37 ^o^C. The cells were incubated with SPIO-FA in the culture medium for 3 days at 37 ^o^C. The cells were washed with PBS solution and fixed with a 1:1 (v/v) mixture of methanol and acetone. After washing with PBS solution, the cell nuclei were stained with propidium iodide (50 mg/mL in PBS solution) for 30 min. Apoptosis-inducing effect of heparan nanogels was evaluated by using a Magic Red caspase detection kit. The cells were examined by using an LSM510 confocal laser scanning microscope (Carl Zeiss, Germany).

**A.Western blot analysis**

Western blot analysis was performed to determine the expression of caspase-3, bax, bcl-2 and survivin protein. Whole cell protein extracts were taken after the incubation of CCRF CEM cells for 72 hours under above-described conditions. Total protein was isolated on ice and subjected to 10% sodium dodecylsulfate polyacrylamide gel electrophoresis (SDS-PAGE) gels using modified radio immuno-precipitation assay buffer, and transferred to a polyvinylidene difluoride membrane (Bio-Rad). Western blotting was performed with a 1:1000–1200 dilutions of monoclonal antibodies against either anti-human caspase-3, bax, bcl-2, survivin or β-actin antibody in 5% nonfat dry milk, and then with horseradish peroxidase-conjugated goat anti-rabbit (1:5000) as a secondary antibody. The bands were detected using an enhanced chemiluminescence detection system (Amersham, Buckinghamshire, UK).

**Statistical Analysis**

All data were presented as means ± standard deviation in triplicate and analyzed using SPSS software (v. 15.0; SPSS Inc., Chicago, IL). The difference among various groups was analyzed by ANOVA test, and P-values of less than 0.05 were considered significant. 

## Results

**A. Morphological characterizations**

**Figure 1:**
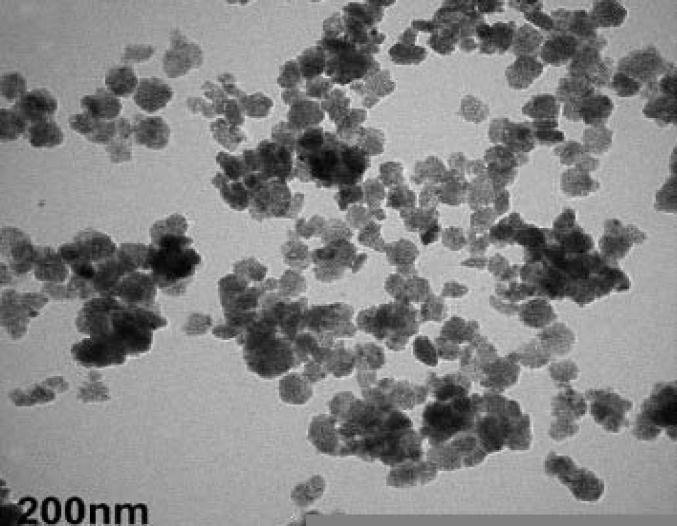
Transmission electron microscopic image of SCIO-FA at 125,000× magnification

TEM image of SPIO-FA was shown in Figure 1. Nanoparticles appeared mono-dispersed spheres with a solid and consistent structure.

**B. Synergistic effect of different concentrations FA on the cytotoxicity of CCRF CEM cells**

The FA concentrations used in this experiment were 10, 30, 50, 70, 100, 150 and 200 µM, where 50 µM FA showed 92% inhabitation rate after 72 hours of incubation of the CCRF CEM cells at 37 ^o^C. 

**Figure 2:**
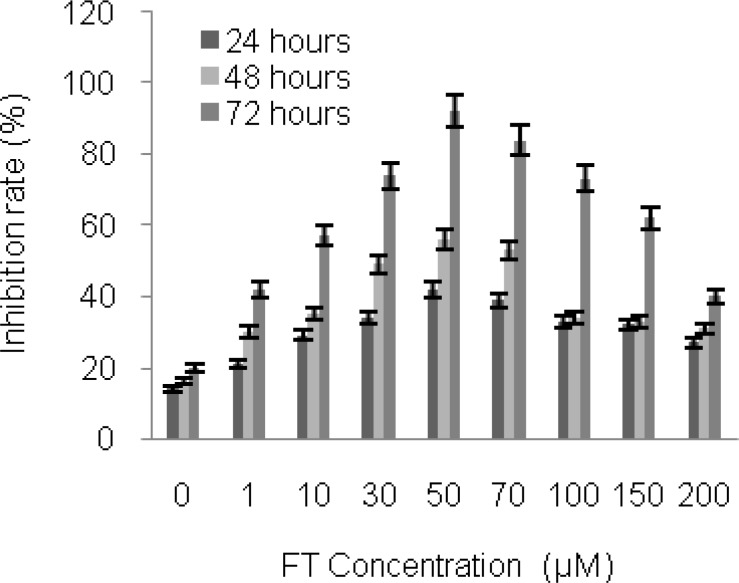
Synergistic effect of different concentrations FA on the cytotoxicity of CCRF CEM cells. FA (10, 30, 50, 70, 100, 150 and 200 µM) was added to the culture medium containing CCRF CEM cells and incubation was done for 72 hours at 37 ^o^C

Furthermore, it was observed that the cancer cell growth inhibition rate was significantly lower in the absence of FA (20%, p<0.05) as compared to the CCRF CEM cells containing 50 µM FA, conjugated to the SCIO-DOX (Figure 2). 

**A. Synergistic effect of FA-SPIO on the apoptosis of CCRF CEM cells**

According to the results shown in Figure 3, 12% of the CCRF CEM cells underwent apoptosis in the presence of 50 µM FA, which was not considerable as compared to the control group (9%; p≥0.05). The apoptosis of CCRF CEM cells, incubated with 10 mg/L SPIO for 72 hours, was 15.8% (p<0.05). While, the combination of SPIO -FA increased the apoptosis up to (40%; p<0.05).

**B. Morphological changes of CCRF CEM Cells**

**Figure 4:**
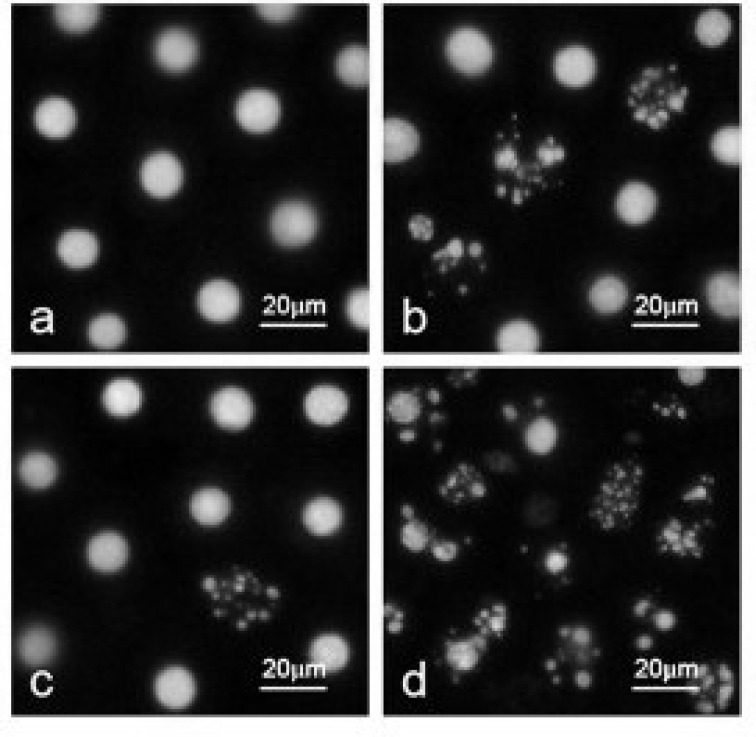
Demonstration of cellular process destruction in the presence of SPIO-FA. a) CCRF CEM Cells grown in PBS, b) cells with 50 µM FA only, c) cells with SPIO only, and d) cells with SPIO-FA

The morphological changes of CCRF CEM cells were observed under the optical microscope. As shown in Figure 4, CCRF CEM cells, in control group, displayed normal and healthy shape, demonstrated by the clear skeletons (Figure 4A). While, after treatment with 10 mg/L SPIO for 72 hours, typical cyto-morphological features of apoptosis in CCRF CEM cells were evident, such as cell shrinkage, chromatin condensation, margination, and the presence of apoptotic bodies (Figure 4B). On the other hand, 50 µM FA caused necrosis in the CCRF CEM cells (Figure 4C). The same, but more significant results were seen when the cells were treated with SPIO-FA complex. While, the cells displayed the typical phenomena of apoptosis including chromatin condensation, nucleolus pyknosis, and nuclear fragmentation in the presence of our modified “anticancer nanomedicine” (Figure 4D). 

**C. Western blot analysis for the determination of caspase-3, bax, bcl-2 and survivin expression**

**Figure 5:**
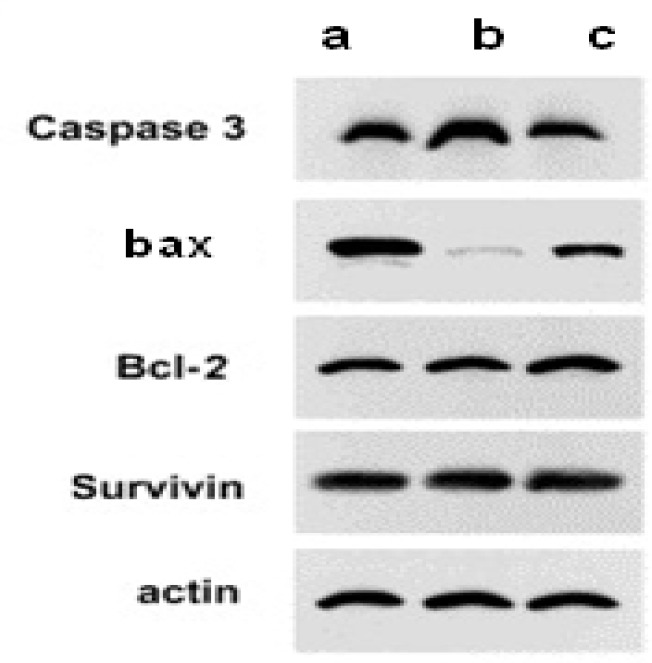
Expression of caspase-3, bax, bcl-2 and survivin protein in CCRF CEM cells by western blot after separate and combined treatment with SPIO, FA and SPIO-FA for 72 hours. β-actin served as a control. CCRF CEM cells were incubated with (a) 10 mg/L SPIO; (b) SPIO with 50 µM FA and (c) with SPIO-FA complex

Our results have shown that the caspase-3, bax, bcl-2 and survivin proteins, in the CCRF CEM cells, treated with 50 µM FA had no significant changes as compared to control group (p<0.05). However, the level of caspase-3 and bax proteins was increased in the presence of SPIO dramatically as compared to control group (p<0.05) (Figure 5). Furthermore, these two kinds of proteins, whose genes were dramatically up-regulated in the presence of SPIO-FA (Figure 5; p< 0.05). Reversely, compared with the control group and the FA-treated group, the level of bcl-2 and caspase was lower, in the presence pf SPIO-FA, as described previously (P-value<0.05).

**Fiure 3:**
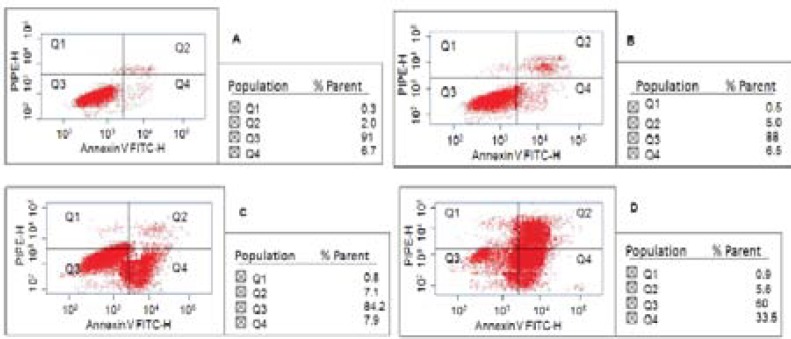
Effect of SPIO-FA on the apoptosis of CCRF CEM cells for 72 hours. A) Control; B) Incubated with 50 µM FA; C) Incubated with 10 mg/L SPIO (Fe_3_O_4_); D) Incubated with SPIO-FA

## Discussion

Although many chemotherapy drugs are used clinically, the overall survival of cancer patients is still unsatisfactory. The majority of chemotherapy medicines have serious adverse effects in addition to their clinical effects. So, there is a serious need to introduce new methods and compounds that prove to be successful fighters of cancer as well as show least side effects.

Biodegradable polymers containing entrapped drug can be placed in the body, and are used for localized drug delivery and/or the controlled release of a drug over a period of months. As the polymer slowly degrades, therapeutic levels of the anti-tumour peptide are maintained for up to 3 months, making the therapy very convenient for patient use (8). Although such polymer-based drug-delivery systems have been important advances, the development of nano-sized vectors enable tumours to be targeted more precisely, the vectors can move around in the body and selectively localize a therapeutic drug payload to metastatic tumours. Conjugation to hydrophilic polymeric carriers can also improve the water solubility of hydrophobic drugs such as doxorubicin and paclitaxel, enabling easier formulation and patient administration. 

For example, it is recently suggested that heparan strongly interferes with the activity of growth factors such as basic fibroblast growth factor (bFGF), thereby inhibiting angiogenesis essential for tumor progression (9). Moreover, heparan is able to attenuate tumor metastasis by blocking selectin-mediated adherence of cancer cells to vascular endothelium or platelets (9). More recent studies showed strong evidence that free heparan molecules internalized within the cells interacted with transcription factors, playing an important role in inducing apoptotic cell death (10, 11). These distinctive functional activities of heparan have significant implications in the development of effective tumour targeted delivery systems, which prompted us to develop heparan based nanoparticulate carriers that could be delivered within tumor cells for apoptotic cell death. The present results prove this fact. 

Most anticancer agents exert their anticancer effects by inducing apoptosis (12). Recently, super paramagnetic iron oxide (Fe_3_O_4_) nanoparticles SPIO-NPs are widely used for targeted drug carriers to enhance the efficiency of anticancer drug delivery based on the ability of target-orientation and sustained-release properties (11). Previous studies have demonstrated the synergistic effect of SPIO nanoparticles with anticancer drugs on the intracellular accumulation in cancer cells (13-15). During the present study, the potential synergistic effects of SPIO-FA were demonstrated while using doxorubicin as the shelled anticancer drug. The present cytotoxic analyses have shown that SPIO-FA caused an increased toxicity in CCRF CEM cells and the presence of SPIO decreased the IC50 of FA in CCRF CEM cells. Effect of present modified nanomedicine was also tested on the apoptosis of CCRF CEM cells. The addition of 10 mg/L SPIO caused a 40% increase in the apoptotic percentage of CCRF CEM cells as compared to those treated with the FA alone (12%) for 72 hours. Our outcomes clearly indicate that a SPIO-FA-based drug delivery system can decrease the IC50 of FA and induced apoptosis in CCRF CEM cells.

In order to check whether the effects of SPIO, along with 50 µM FA was different from that of FA alone, we demonstrated that the CCRF CEM cells, incubated with 50 µM FA and 10 mg/L SPIO for 72 hours, showed a typical morphological features of apoptosis under the optical microscope, while 50 µM FA led cells to necrosis. These results suggest that a combination of SPIO and FA could be a feasible candidate in the development of anticancer drugs. Apoptosis is the consequence of a series of precisely regulated events that are frequently altered in tumor cells. In general, the sequence of events has been broadly categorized into two pathways: the extrinsic pathway, which involves the activation of the tumor necrosis factor (TNF)/Fas death receptor family and the intrinsic pathway, which involves the mitochondria. In both pathways, an apoptotic death stimulus results in the activation of caspases, the major executioners of this process, either directly or via activation of the mitochondrial death program (16, 17). 

It is well known that caspase-3 is the most characterized effector caspase, and its activation leads to the final stages of cellular death by proteolytic dismantling of a large variety of cellular components on one hand, and activation of proapoptotic factors on the other hand (16, 17). Our study showed that FA combined with SPIO dramatically upregulated the transcription and expression of caspase-3 in CCRF CEM cells. This result supports the promotion of FA-induced apoptosis by SPIO, which was related to the level of genes and proteins expression. In tumor cells, apoptosis can be induced either by activation of molecules upstream of apoptosis signaling or by inhibition of antiapoptotic factors. 

Survivin, a member of the inhibitor of apoptosis protein (IAP) family, is overexpressed in virtually every human cancer. In several tumor cell lines, the presence of survivin correlates with resistance to apoptosis and is associated with increased malignancy (18). Previous *in vitro *studies showed that inhibition of survivin restored or enhanced the cytotoxicity of chemoreagents (19), and animal studies showed a superb efficacy against xenografts using an adenoviral strategy targeted to surviving (20). At present, survivin is validated as a cancer therapeutic target (21). Our data showed that the expression of antiapoptotic genes such as bcl-2, survivin of CCRF CEM cells were significantly down-regulated after co-treatment of FA with SPIO, whereas the expression of bax was up-regulated. Bax and bcl-2 both belong to the bcl-2 family (22). 

Overexpression of Bax has been shown to accelerate cell death (26), while that of antiapoptotic proteins such as bcl-2 represses the death function of bax (24). Thus, the ratio of bcl-2/bax might be a critical factor of a cell’s threshold for undergoing apopto sis (25).Although bcl-2 and survivin are both apoptosis inhibitors, they work through different pathways in the regulation of cell apoptosis. The antiapoptotic protein bcl-2 mainly inhibits the mitochondrial pathways (26), while survivin directly blocks the processing and activation of effector caspase-3 and caspase-7, which commonly acts downstream of both apoptosis signaling pathways, which suggests that SPIO loaded with FA induced cell apoptosis through various pathways.

## Conclusion

Our study demonstrates for the first time that SPIO can promote apoptosis induction of FA *in vitro *in CCRF CEM cells, and the synergistic effect of that composite on apoptosis induction may owe to the regulation of various proliferative and antiapoptotic gene products, including caspase-3, bax, bcl-2 and survivin. Thus, it may be possible that a combination of SPIO and FA is sufficient and less toxic method in blood cancer therapy.
